# Screening of wild-type *Saccharomyces cerevisiae* strains for single-cell protein production in white grape juice

**DOI:** 10.1007/s10068-026-02215-8

**Published:** 2026-06-30

**Authors:** İpek Ceren Yeşildağ, Remziye Yılmaz

**Affiliations:** FoodOmics Laboratory, Department of Food Engineering, Hacettepe University, 06800 Ankara, Türkiye

**Keywords:** Single-cell protein, Grape juice, *Saccharomyces cerevisiae*, Screening, Biomass production

## Abstract

Increasing demand for sustainable protein sources has intensified interest in single-cell protein (SCP) production. This study evaluated the fermentation performance of wild-type *Saccharomyces cerevisiae* strains isolated from grape vineyards to identify candidates for SCP bioprocesses. Following morphological characterization and pH screening, strains were cultivated in white grape juice supplemented with 1% (w/v) yeast extract for 72 h at 28 °C. K-means clustering, principal component analysis (PCA), and growth curve modelling were applied to differentiate the strains. Strains HUF16M2K10004 and HUF16M3G11088 reached wet cell weights of 21.56 ± 0.13 and 27.90 ± 0.49 g/L, dry cell weights of 6.01 ± 0.05 and 6.46 ± 0.44 g/L, water-soluble protein contents of 2.00 ± 0.15 and 1.92 ± 0.07 g/L, with growth rates of 0.15 ± 0.02 and 0.23 ± 0.02 h⁻^1^, comparable to those of the commercial reference strains. Both wild-type strains represent preliminary candidates for future SCP-oriented characterization studies.

**Supplementary Information:**

The online version contains supplementary material available at 10.1007/s10068-026-02215-8.

## Introduction

Yeasts, particularly *Saccharomyces cerevisiae* (*S. cerevisiae*)*,* have played a pivotal role in fermentation and food production for centuries. Today, *S. cerevisiae* remains a widely used model organism with GRAS (Generally Recognized as Safe) status and is integral to numerous industrial applications, from small-scale processes to large-scale production (Stewart, 2014). Owing to its robust fermentative capacity and resilience to environmental stresses, including low pH, elevated ethanol concentrations, and nutrient limitations (Stewart, 2014), *S. cerevisiae* is extensively employed in the production of fermented foods, food ingredients, probiotics, bioethanol, biopharmaceuticals, and single-cell protein (Anderson et al., 2024; Khalid et al., 2026).

Growing global concerns regarding food security and sustainability have renewed interest in yeast biomass as an alternative protein source (Gervasi et al., 2018). Single-cell proteins (SCP) produced using bacteria, yeasts, and algae have attracted considerable attention due to their ability to generate biomass with protein contents reaching up to 80% (Bratosin et al., 2021). Among these sources, *Saccharomyces cerevisiae* is widely utilized for SCP production due to its balanced nutritional composition, including proteins (33–54%), essential amino acids, fats, carbohydrates, vitamins, and minerals, which collectively support its suitability for food and feed applications (Jach et al., 2022). Moreover, its ability to utilize diverse agro-industrial residues enhances its value in sustainable fermentation systems and waste valorisation strategies (Gervasi et al., 2018). Accordingly, the identification of strains with favourable biomass yield and growth performance is critical for the development of efficient and sustainable fermentation processes.

Although commercial and genetically modified yeast strains dominate industrial fermentations, wild-type *S. cerevisiae* strains offer distinct advantages due to their inherent genetic diversity and adaptability to environmental stresses (Parapouli et al., 2020). These strains may exhibit improved growth kinetics or biomass yields, creating new opportunities for alternative protein production and sustainable fermentation technologies. Consequently, recent research has increasingly focused on screening wild-type *S. cerevisiae* strains for a wide range of biotechnological applications (Basa et al., 2022; Moreira-Ramos et al., 2024).

While various culture media have been explored for strain screening, further research is required to assess yeast performance in fermentation substrates that replicate the physicochemical conditions and selective pressures encountered in natural fermentation environments. In this context, grape juice represents a physiologically relevant substrate, as it provides fermentable sugars, organic acids, essential nutrients, and selective stresses characteristic of natural fermentations. Its complex composition enables effective discrimination of strain performance, making it well-suited for evaluating growth behaviour and biomass formation in *S. cerevisiae* (Bovo et al., 2018).

Building on this rationale, the present study aimed to conduct a preliminary screening of wild-type *S. cerevisiae* strains, alongside commercial strains, in white grape juice as a food-grade screening medium. For this purpose, fermentation performance, growth kinetics, biomass yield, and water-soluble protein (WSP) content were evaluated to identify preliminary strains for future single-cell protein applications (Yeşildağ, 2024).

## Materials and methods

### Strains and culture conditions

Wild-type *Saccharomyces cerevisiae* strains, previously isolated from grape vineyards in various regions of Türkiye, were obtained from the Hacettepe University FoodOmics Laboratory Culture Collection (HUFCC), which is registered in the World Data Centre for Microorganisms database (WDCM#1325). The strains were stored at -80 °C in 50% (v/v) Yeast Peptone Dextrose (YPD) Broth (Sigma Aldrich, St. Louis, USA) containing 25% (v/v) glycerol. Relevant strain information, including isolation codes and NCBI accession numbers, is summarized in Table 1.

**Table 1:** Commercial and wild–type *Saccharomyces cerevisiae* strains from HUFCC–WDCM#1325 used in this study, including isolation codes, NCBI accession numbers

Strain name	Isolation code	NCBI accession no	Origin
*S. cerevisiae* ATCC 9763	9763	OQ876808	Commercial
*S. cerevisiae* MERIT*™* *(Chr. Hansen)*	HUF16M1C0004	PV819393.1	Commercial
*S. cerevisiae* ATCC 6328	6328	OQ912898	Commercial
HUF	16M2K10004	OQ912899	–
HUF	16M3B11021	OQ876789	39°45′45.9″N 33°30′59.5″E
HUF	16M3C11032	OQ876790	39°05′02.8″N 34°22′50.3″E
HUF	16M3C11033	OQ876791	39°05′02.8″N 34°22′50.3″E
HUF	16M3G11088	OQ876792	39°48′12.8″N 30°35′36.9″E
HUF	16M3H11101	OQ876793	40°42′09.1″N 33°31′22.6″E
HUF	17M3C31063	OQ876795	39°27′26.1″N 33°57′37.1″E
HUF	17M3D31088	OQ876799	38°34′22.9″N 34°36′59.8″E
HUF	17M3E21113	OQ876802	38°32′15.3″N 34°23′52.2″E
HUF	17M3E21114	OQ876803	38°32′15.3″N 34°23′52.2″E
HUF	17M3F21122	OQ876804	40°07′25.2″N 33°02′13.9″E
HUF	17M3H21208	–	37°19′15.9″N 33°19′46.4″E
HUF	17M3H21209	–	37°19′15.9″N 33°19′46.4″E
HUF	18M2Y10013	–	–
HUF	18M2Z10001	–	–

*S. cerevisiae* ATCC 9763, *S. cerevisiae* MERIT™ (Chr. Hansen), and *S. cerevisiae* ATCC 6328 were commercially obtained. All yeast strains were inoculated into YPD Broth medium and revived by incubating at 28 °C for 24 h. Subsequently, the strains were cultivated on Yeast Extract Peptone Dextrose (YPD) Agar (Sigma Aldrich, St. Louis) at 28 °C for strain growth and maintenance.

### Verification of the morphological characterization of yeast strains

Morphological characterization and verification were performed on the yeast strains listed in Table 1. Strains were cultivated on YPD Agar (Sigma Aldrich, St. Louis) at 28 °C for 72 h. Following incubation, the Petri dishes were photographed to verify the macroscopic morphology of typical *S. cerevisiae* yeast colonies. A single colony was then subjected to methylene blue staining for analysis (Painting and Kirsop, 1990). The stained colony was subsequently examined under a light microscope (Olympus LS, Tokyo, Japan) at ×100 magnification to assess its morphological features.

### Growth at different pH

To identify acid–tolerant candidates for subsequent fermentation trials, a preliminary qualitative screening was performed. Single colonies from pure cultures of each strain listed in Table 1, which were maintained on YPD Agar (Sigma Aldrich, St. Louis, USA), were separately inoculated into 2 mL of YPD Broth (Sigma Aldrich, St. Louis, USA) at pH 6.5, and incubation was performed at 28 °C for 24 h. To evaluate strain growth under acidic conditions, the procedure was repeated with the medium adjusted to pH 3.3 using 1 N HCl (Sigma Aldrich, St. Louis, USA). After incubation, growth at both pH values was assessed qualitatively based on visible turbidity formation in the culture tubes and classified as robust visible growth (+ +) or weak visible growth ( +).

### Phylogenetic analysis

Phylogenetic analysis based on the large subunit ribosomal RNA (LSU; 25S/28S rRNA) gene sequences was performed to molecularly characterize the wild–type isolates and to evaluate their evolutionary relationship with commercial reference strains.

Partial LSU sequences of four isolates—*Saccharomyces cerevisiae* ATCC 9763 (HUF16M1C0002; OQ876808.1), HUF16M2K10004 (OQ912899.1), HUF16M3G11088 (OQ876792.1), and HUF16M3H11101 (OQ876793.1)—were obtained by Sanger sequencing and retrieved from the NCBI GenBank database. Although the commercial *S. cerevisiae* MERIT™ strain was included in the experiments, its ribosomal DNA sequence was not publicly available. Therefore, the well–characterized reference strain *S. cerevisiae* S288C (NR_132209.1) was used to provide a standardized phylogenetic framework. The full–length 25S rRNA sequence of S288C was aligned with the partial LSU sequences, and only the overlapping homologous regions were retained to ensure positional comparability.

Multiple sequence alignment was performed using the ClustalW algorithm implemented in MEGA11 software. Phylogenetic relationships were inferred using the Neighbour–Joining (NJ) method. Evolutionary distances were calculated using the Tamura 3–parameter (T92) model. The robustness of the inferred topology was assessed by bootstrap analysis with 1000 replicates. All ambiguous positions were eliminated using the pairwise deletion option. The final dataset comprised 911 aligned nucleotide positions.

The analysis included seven nucleotide sequences: five *S. cerevisiae* strains and two non–*Saccharomyces* reference species (*Kluyveromyces lactis* AJ229055 and *Zygosaccharomyces rouxii* AJ229050) used as outgroups. All evolutionary analyses were conducted in MEGA11.

### Growth measurement

Prior to the screening of yeast strains for biomass yield and water-soluble protein (WSP) content, a primary investigation was conducted to determine growth characteristics. The inoculum was prepared by transferring two loopfuls of yeast strain from YPD agar to 10 mL white grape juice (Kavaklıdere Wines Co., Ankara, Türkiye) supplemented with 1% (w/v) yeast extract as a nitrogen source and incubated at 28 °C for 24 h. After incubation, 100 mL white grape juice medium was inoculated with preculture 1% (v/v) and incubated at 28 °C for 24 h. Periodic sampling was performed to monitor yeast growth and the calculation of growth kinetics.

### Screening of yeast strains

The screening of yeast strains was performed using white grape juice (Kavaklıdere Wines Co., Ankara, Türkiye) supplemented with 1% (w/v) yeast extract (Lab M, Lancashire, United Kingdom) as a nitrogen source to compensate for the inherently low yeast assimilable nitrogen content of grape juice and to ensure uniform nitrogen availability across all strains during comparative fermentation. In this regard, 100 mL of fermentation medium was sterilized at 121 °C for 15 min.

The inoculum was prepared as described above. 100 mL white grape juice medium was inoculated with preculture 1% (v/v) and incubated at 28 °C for 72 h. Samples were collected to determine biomass yield and water–soluble protein (WSP) content during fermentation.

### Biomass purification and characterization

After fermentation, liquid samples were subjected to centrifugation at 6,000×g for 5 min using a NF-800 centrifuge (Nüve, Ankara, Türkiye) and then washed twice with sterile distilled water. Subsequently, the collected wet biomass was weighed and expressed as wet cell weight (WCW) in g/L of broth. Dry cell weight (DCW) was determined by lyophilizing (Christ Alpha 1–4 LD plus freeze dryer, Osterode am Harz, Germany) the wet biomass for 24 h. After lyophilization, the dried biomass was weighed, and the value was expressed as g/L of broth. Water–soluble protein (WSP) content of the yeast biomass was determined using the Bradford method (Bradford, 1976). Lyophilized biomass was resuspended in distilled water and subjected to vortex mixing for 1 min, and the supernatant was collected following centrifugation at 6,000×g for 5 min. A 50 µL aliquot of each sample was mixed with 250 µL Bradford reagent and incubated for 10 min at room temperature, and absorbance was measured at 595 nm. A calibration curve was constructed using bovine serum albumin (BSA; Sigma–Aldrich, St. Louis, MO; Cat. No. A2153) at concentrations ranging from 0 to1 mg/mL. Protein values were corrected against a cell–free fermentation medium blank to account for potential interference from yeast extract–derived components. WSP content was expressed as g protein per liter of fermentation broth (g/L).

### Calculation of growth kinetics

Yeast growth was monitored by optical density (OD) measurements using a spectrophotometer (Genesys 10S UV–VIS, Thermo Scientific) at 600 nm. The Dashing Growth Curves application (Reiter and Vorholt, 2024) was used to calculate specific cell growth rate (h^−1^) and doubling time (h) values. The growth kinetics of the yeast strains were calculated based on optical density (OD_600_) values measured during the exponential phase of growth.

### Statistical analysis

The statistical analyses were performed using SPSS Statistics Version 23 (IBM) software (IBM Corp., Armonk, NY, USA). All the experiments were conducted in biological duplicates, and the results are shown as the mean value ± standard deviation (SD). Optical density (OD_600_) and water–soluble protein (WSP) content measurements were obtained from two independent biological replicates, each analyzed in technical duplicates. Dry cell weight (DCW), wet cell weight (WCW), and growth kinetics measurements were determined in triplicate as technical replicates from a single experimental batch. Differences in OD₆₀₀ and WSP data were evaluated by one–way analysis of variance (ANOVA), followed by the Tukey’s post–hoc test with a 95% confidence level (P < 0.05), whereas WCW, DCW, and growth kinetics data are reported as descriptive results.

Cluster analysis (K–means) and principal component analysis (PCA) were performed in R version 4.4.3 via RStudio (v. 2025-02-28, Boston, MA, USA), using the fermentation data of the yeast strains (Supporting Information Table S3). K–means clustering analysis and PCA were conducted using the “FactoMineR” package (Lê et al., 2008). PCA visualizations were generated with the “factoextra” package (Kassambara and Mundt, 2020 ), while K–means clustering results were visualized using the dplyr, tidyr, and ggplot2 packages (Wickham, 2016; Wickham et al., 2023, 2024).

## Results and discussion

### Morphological characteristics of yeast strains

Previously isolated wild–type yeast strains (Table 1), together with commercial strains (*S. cerevisiae* ATCC 9763, *S. cerevisiae* ATCC 6328 and *S. cerevisiae* MERIT*™*), were morphologically characterized and verified in terms of colony characteristics. Colony morphology, including color, appearance, and texture, was assessed on YPD Agar following incubation at 28 °C for 72 h. A comprehensive overview of the macroscopic and microscopic morphological features of all strains is provided in Supporting Information Table S1.

Upon examination of the macroscopic morphologies of the strains, it was observed that while some of the strains exhibited an oval, smooth, and light cream–colored appearance, others displayed an oval, shiny, and light cream–colored morphology (Supporting Information Table S1). Notably, strain HUF18M2Z10001 displayed a smaller colony radius compared to the other strains. These macroscopic characteristics are consistent with previously reported morphological features of *S. cerevisiae* (Haile and Kang, 2019; Stewart, 2014).

Microscopic examination revealed that the majority of strains exhibited an ovoid cell morphology, while some displayed an ellipsoidal shape, in agreement with earlier reports (Abdulla et al., 2022; Guadalupe-Daqui et al., 2021). It has been found that the strain HUF18M2Z10001 exhibits a considerably smaller colony appearance in comparison to the other strains, as evidenced by its macroscopic morphology. Following morphological verification, all strains were subjected to growth assessment under acidic conditions to evaluate acid tolerance as a physiologically relevant selection criterion for subsequent fermentation.

### Growth at different pH

The pH of the culture medium exerts a significant influence on yeast growth and fermentation performance, thereby directly affecting biomass production (Liu et al., 2015). To evaluate the acid tolerance of the strains, growth was initially assessed in YPD broth at neutral pH (6.5) and subsequently under acidic conditions (pH 3.3). As shown in Supporting Information Table S1, most strains exhibited robust growth at pH 6.5; however, notable differences were observed under acidic conditions.

Among the tested isolates, the commercial strains 9763 and MERIT™, which are widely used in fermented beverage production, demonstrated the expected high adaptability to low pH. In contrast, the commercial strain ATCC 6328 did not exhibit visible growth at pH 3.3 (Supporting Information Table S1) and was therefore excluded from subsequent fermentation trials. Notably, three wild–type yeast strains—HUF16M2K10004, HUF16M3G11088, and HUF16M3H11101—also maintained strong growth at pH 3.3, comparable to the commercial reference strains. The observed acid tolerance of these wild–type yeast strains is consistent with their ecological origin, as yeasts associated with grape surfaces are frequently exposed to acidic environments and are subject to natural selection for acid–resilient phenotypes (Wang et al., 2022). Acid tolerance is a critical trait for SCP production, as many food–grade substrates —particularly fruit–derived media—naturally exhibit low pH and therefore require strains capable of sustaining growth under such conditions. Accordingly, the five acid–tolerant strains were selected for molecular identification to confirm species assignment before comparative fermentation trials in white grape juice.

### Phylogenetic analysis

**Fig. 1:**
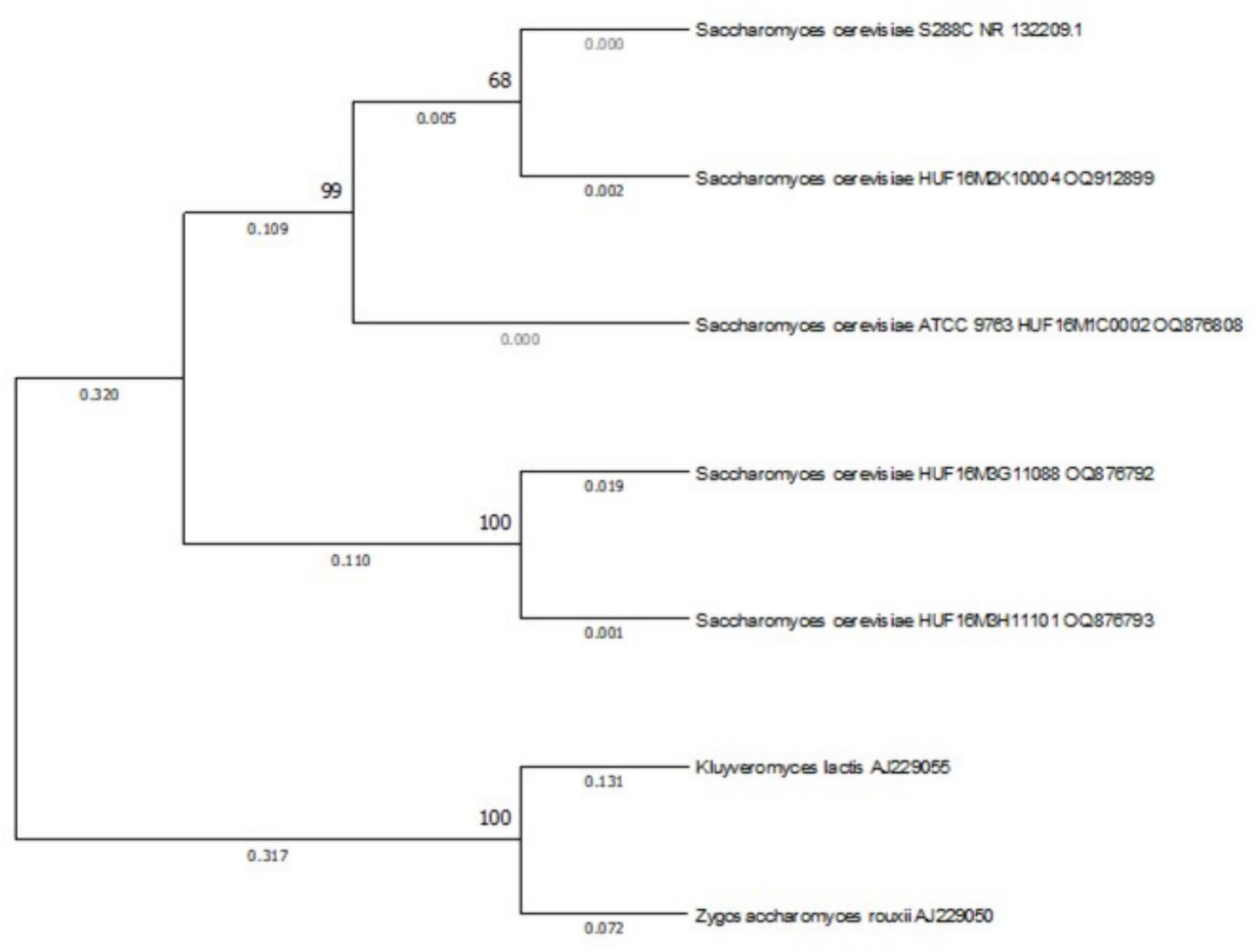
Phylogenetic tree based on LSU (25S/28S rRNA) gene sequences of commercial and wild–type *Saccharomyces cerevisiae* strains

Phylogenetic analysis based on partial LSU (25S/28S rRNA) sequences was performed to confirm the species–level identification of isolates and revealed that all analyzed isolates clustered within the *Saccharomyces cerevisiae* clade (Fig. 1). The *S. cerevisiae* strains were clearly separated from the non–*Saccharomyces* outgroups (*Kluyveromyces lactis* and *Zygosaccharomyces rouxii*) with strong bootstrap support (100%), confirming the taxonomic identity of the studied isolates.

Within the *S. cerevisiae* cluster, two distinct subclades were observed. The first subclade grouped the reference strain S288C with wild–type strain HUF16M2K10004 (bootstrap 68%), while ATCC 9763 (HUF16M1C0002) formed a closely related lineage within the same major branch (bootstrap 99%). The short branch lengths (0.000–0.005 substitutions per site) indicate a high degree of sequence similarity among these strains in the LSU region.

The second subclade consisted of the wild–type isolates HUF16M3G11088 and HUF16M3H11101, supported by a bootstrap value of 100%. The minimal genetic distance between these two strains (0.001 substitutions per site) indicates a high degree of sequence similarity within the LSU rRNA gene region and supports their classification within the *S. cerevisiae* species. These results confirm that all analyzed isolates belong to *S. cerevisiae* while the limited intraspecific variation observed within the LSU region reflects the well–recognized constraint of this marker for strain–level resolution. Based on the confirmed species identity and the acid tolerance profiles described above, the selected strains were subsequently evaluated for growth and biomass production in white grape juice medium to assess their fermentation performance under conditions relevant to single–cell protein applications.

### Growth measurement

**Fig. 2:**
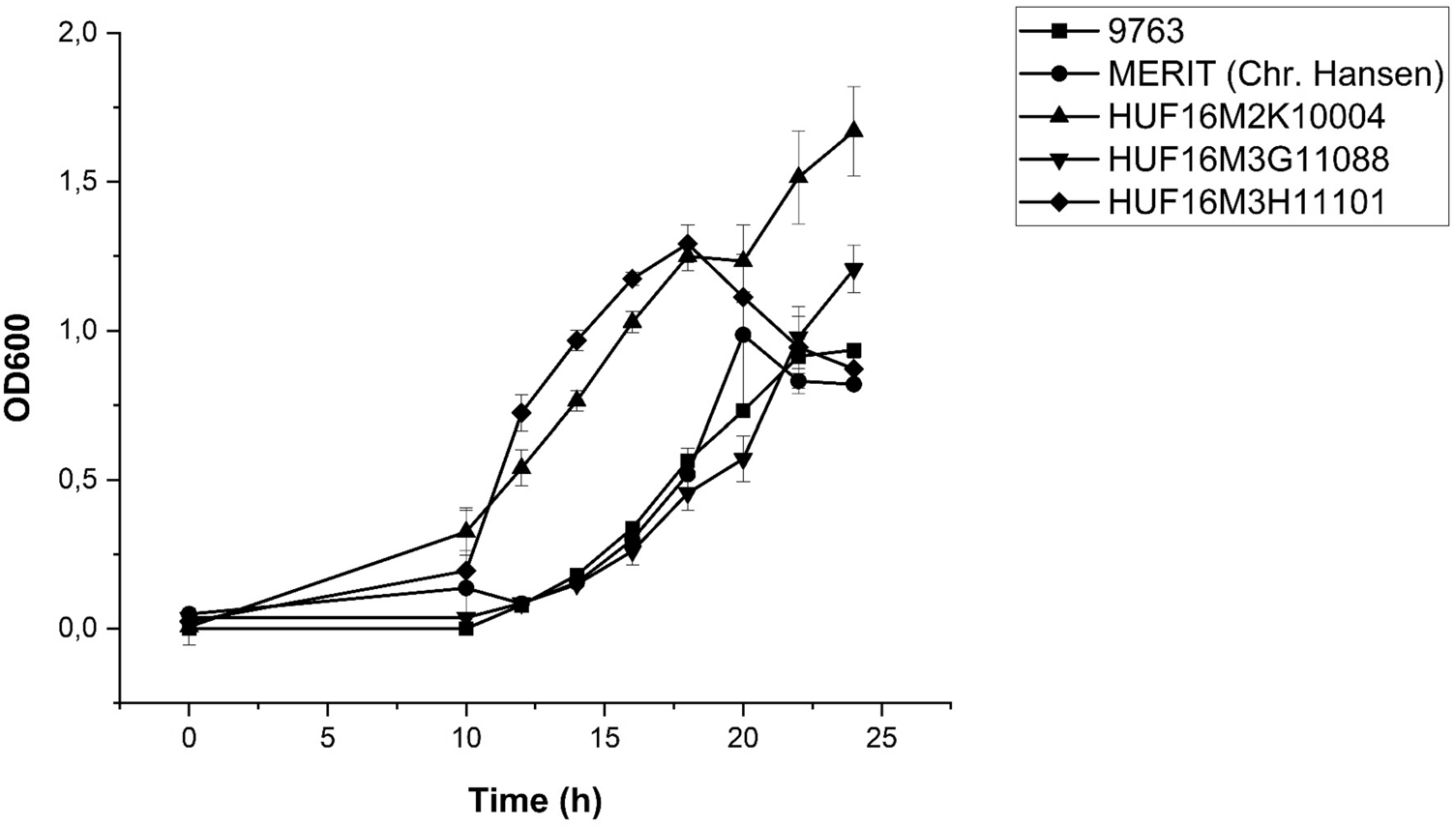
Growth curves of *S. cerevisiae* 9763, MERIT^TM^, HUF16M2K10004, HUF16M3G11088, and HUF16M3H11101 strains determined by optical density measurement (OD_600_)

A preliminary fermentation was conducted at 28 °C for 24 h to evaluate the growth profiles of the pre–selected *Saccharomyces cerevisiae* strains 9763, MERIT™, HUF16M2K10004, HUF16M3G11088, and HUF16M3H11101 in white grape juice medium supplemented with 1% (w/v) yeast extract (Fig. 2). Strains 9763 and HUF16M3H11101 exhibited a lag phase of approximately 0–10 h, whereas MERIT™ and HUF16M3G11088 required up to 14 h to adapt to the juice matrix. During this adaptation period, no significant changes in cell concentration were observed (p > 0.05), indicating limited growth during the lag phase (Supporting Information Table S2). In contrast, HUF16M2K10004 exhibited the shortest lag phase (< 10 h), suggesting rapid adaptation to the white grape juice environment.

The logarithmic phase was observed between 12–22 h, 14–20 h, 10–24 h, 14–24 h, and 10–18 h for 9763, MERIT™, HUF16M2K10004, HUF16M3G11088, and HUF16M3H11101, respectively. The commercial strains 9763 and MERIT™ demonstrated a stationary phase after 22 h and 20 h, respectively. In contrast, HUF16M3H11101 entered the death phase after 18 h.

Based on these findings, it can be concluded that 9763 and MERIT™, as well as HUF16M2K10004 and HUF16M3G11088, showed similar growth profiles. Overall, these four strains demonstrated comparable growth performance, whereas HUF16M3H11101 showed markedly reduced growth, reaching a final OD_600_ of 0.87 ± 0.04 at 24 h, compared with an average OD_600_ of 1.16 among the other strains. Similarly, Stivala et al. (2017) reported an approximate maximum OD_600_ of 1.5 after 96 h of fermentation using grape juice medium supplemented with 10 g/L yeast extract. Notably, despite employing a higher sugar concentration (200 g/L) in their synthetic molasses medium, Eliodório et al. (2023) reported a relatively lower OD_600_ range (0.5–0.8) after 20 h, whereas the white grape juice medium used in the present study, containing 160 g/L total sugars, supported higher cell densities.

### Screening of yeast strains

**Fig. 3:**
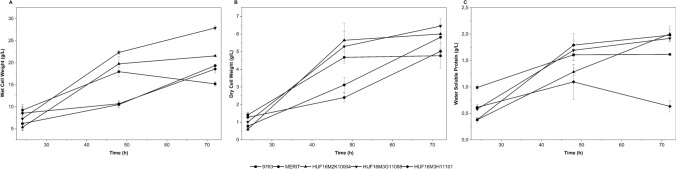
Biomass yields in terms of wet cell weight (**A**), dry cell weight (**B**), and water–soluble protein content (**C**) of 9763, MERIT^TM^, HUF16M2K10004, HUF16M3G11088, and HUF16M3H11101 strains in white grape juice medium

Biomass yield, expressed as wet cell weight (WCW) and dry cell weight (DCW), water–soluble protein (WSP) content, and growth kinetics of the selected strains were monitored during 72 h of fermentation in white grape juice medium (Fig. 3). An overall increase in biomass yield (Fig. 3A and B) and water-soluble protein content (Fig. 3C) was observed as fermentation progressed. During the initial 24 h, WCW and DCW values were comparable across strains (Supporting Information Table S3); however, significant differences were observed in WSP, with 9763 exhibiting highest WSP content (0.99 ± 0.05 g/L), followed by HUF16M3G11088 (0.58 ± 0.01 g/L) and HUF16M3H11101 (0.62 ± 0.05 g/L) respectively, while MERIT™ (0.38 ± 0.04 g/L) and HUF16M2K10004 (0.38 ± 0.04 g/L) showed lowest values, reflecting the early adaptation phase described previously.

After 48 h of fermentation, HUF16M3G11088 exhibited WCW, DCW, and WSP values comparable to those of the commercial strains 9763 and MERIT™, reaching 22.34 ± 0.46 g/L WCW, a DCW of 5.30 ± 0.84 g/L and 1.69 ± 0.19 g/L WSP. Similarly, HUF16M2K10004 achieved a WCW of 19.75 ± 1.65 g/L, a DCW of 5.65 ± 0.95 g/L and a WSP of 1.29 ± 0.07 g/L. In contrast, HUF16M3H11101 exhibited the lowest biomass production, with WCW, DCW, and WSP values of 10.69 ± 0.77, 2.40 ± 0.26 g/L, and 1.10 ± 0.33 g/L, respectively.

At 72 h, HUF16M3G11088 achieved the highest biomass yields, reaching 27.90 ± 0.49 g/L WCW and 6.46 ± 0.44 g/L DCW, along with a WSP content of 1.92 ± 0.07 g/L. This strain also exhibited a notably higher specific growth rate of 0.23 ± 0.02 h^–1^ and a shorter doubling time of 3.08 ± 0.23 h compared to other wild–type strains. Simultaneously, HUF16M2K10004 reached a maximum WCW of 21.56 ± 0.13 g/L, DCW of 6.01 ± 0.05 g/L and a WSP content of 2.00 ± 0.15 g/L. These values exceed many previously reported yields for fungal and yeast strains cultivated on fruit–based substrates, where biomass yields typically range from 0.64 to 2.52 g/L and protein contents from 0.02 to 1.82 g/L (Chakraborty and Bhowal, 2023). Moreover, the observed DCW values are comparable to those reported for scaled–up fermentation systems, in which dry cell weights of up to 8.0 g/L have been achieved (Luyt et al., 2021). HUF16M3H11101 exhibited an increase in biomass at 72 h, with WCW and DCW values of 18.58 ± 0.89 g/L and 5.02 ± 0.12 g/L, respectively; however, a marked reduction in WSP content (0.63 ± 0.11 g/L) was observed. This decrease is likely attributable to yeast autolysis during the death phase (Fig. 2) and nutrient depletion, as previously reported (Alexandre and Guilloux-Benatier, 2006; Dunuweera et al., 2021). Although HUF16M3G11088 and HUF16M3H11101 clustered closely within the LSU–based phylogenetic analysis, they exhibited different fermentation behaviours under the tested conditions. The commercial strain MERIT™, widely used in wine fermentation, demonstrated an increase in WCW and DCW, reaching 19.35 ± 0.26 g/L and 5.82 ± 0.71 g/L, respectively, with a WSP content of 1.98 ± 0.10 g/L. Additionally, a specific growth rate of 0.31 ± 0.06 h^–1^ and a doubling time of 2.36 ± 0.51 h were observed. Similarly, the commercial strain 9763 exhibited an increase in WCW (15.21 ± 0.57 g/L) and DCW (4.77 ± 0.70 g/L), with a specific growth rate of 0.29 ± 0.02 h^–1^ and a doubling time of 2.39 ± 0.15 h. However, its WSP content remained relatively constant at 1.61 ± 0.11 g/L.

Overall, after 72 h of fermentation at 28 °C, WCW, DCW, and WSP content across all strains ranged from 15.21 to 27.90 g/L, 4.77 to 6.46 g/L, and 1.62 to 2.00 g/L, respectively. Notably, the wild–type strains HUF16M2K10004 and HUF16M3G11088 demonstrated biomass yields and WSP contents comparable to those of commercial strains.

### Multivariate analysis of yeast strains

**Fig. 4:**
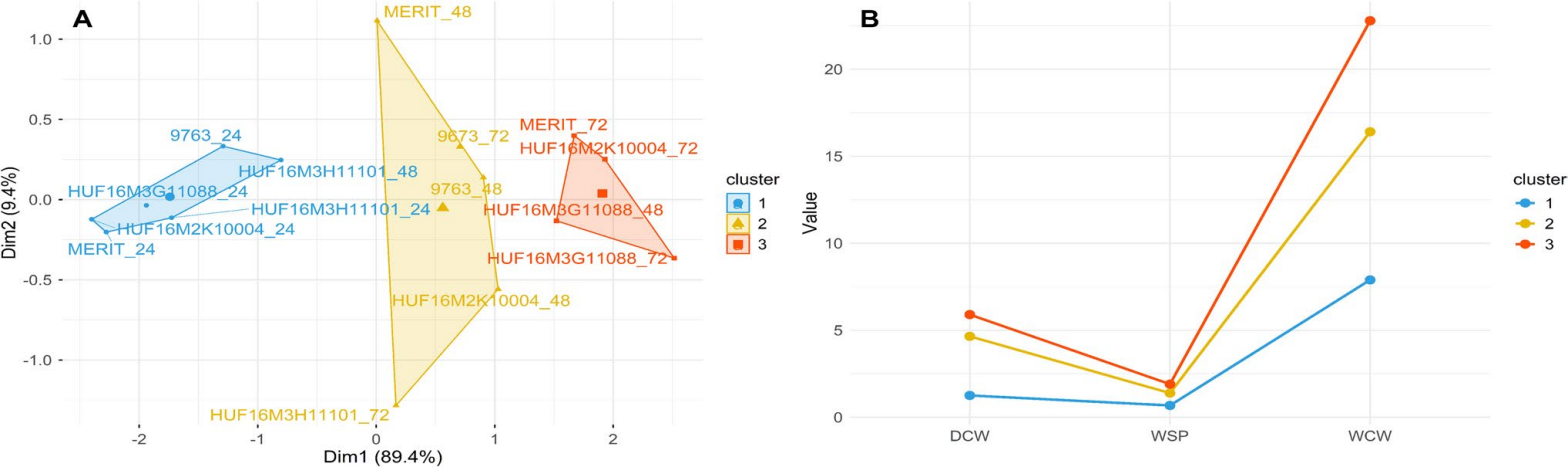
K–means clustering of fermentation parameters (*WCW* wet cell weight, *DCW* dry cell weight, and water–soluble protein content) of *S. cerevisiae* strains 9763, MERIT™, HUF16M2K10004, HUF16M3G11088, and HUF16M3H11101 in white grape juice medium at different incubation times (24, 48, and 72 h). **A** K–means clustering reveals three distinct groups based on fermentation performance; **B** Mean values of fermentation parameters for each cluster

To explore the variation in fermentation performance of five yeast strains, multivariate analyses combining principal component analysis (PCA) and K–means clustering were conducted using a dataset (Supporting Information Table S3 and Table S4) comprising wet cell weight (WCW), dry cell weight (DCW), and water–soluble protein (WSP) content measured at 24, 48, and 72 h of incubation (Fig. 4).

K-means clustering classified the samples into three distinct clusters (Fig. 4A). Cluster 1 comprised samples corresponding primarily to the early stage of fermentation (24 h), characterized by the lowest WCW, DCW, and WSP values. Notably, measurements for HUF16M3H11101 at both 24 h (8.55 ± 1.40 g/L WCW; 1.30 ± 0.30 g/L DCW; 0.62 ± 0.05 g/L WSP) and 48 h (10.69 ± 0.77 g/L WCW, 2.40 ± 0.26 g/L DCW and 1.10 ± 0.33 g/L WSP) were assigned to this cluster, indicating slower biomass and water-soluble protein production compared to other strains. Cluster 2 predominantly included data points from 48 h, characterized by increased WCW, DCW, and WSP content relative to Cluster 1. In addition, both the 48 h and 72 h measurements of strains 9763 and HUF16M3H11101 were grouped within this cluster, suggesting that although biomass accumulation continued over time, it was not accompanied by a proportional increase in WSP content. Cluster 3 primarily included samples collected at 72 h from strains MERIT™, HUF16M2K10004, and HUF16M3G11088, which exhibited the highest WCW, DCW, and WSP content. Within this cluster, HUF16M2K10004 and HUF16M3G11088 reached WCW values of up to 27.90 ± 0.49 g/L and DCW values of up to 6.46 ± 0.44 g/L, and WSP contents of up to 2.00 ± 0.15 g/L, comparable to those achieved by the commercial strain MERIT™. The mean values presented in Fig. 4B further support the greater biomass production and WSP content of strains grouped in Cluster 3.

**Fig. 5:**
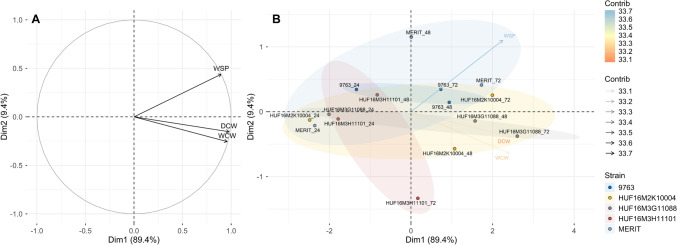
Principal component analysis (PCA) of fermentation parameters (*WCW* wet cell weight, *DCW* dry cell weight, and *WSP *water–soluble protein content) of *S. cerevisiae* strains 9763, MERIT™, HUF16M2K10004, HUF16M3G11088, and HUF16M3H11101 in white grape juice medium at different incubation times (24, 48, and 72 h). **A** PCA biplot of fermentation parameters; **B** PCA showing the contribution of each observation to the variance

In parallel, PCA was conducted using the same dataset to further elucidate relationships among the fermentation parameters (Fig. 5). The first two principal components (PC1 and PC2) together accounted for 98.8% of the total variance. The loading plot (Fig. 5A) indicates that WCW, DCW, and WSP content were all positively correlated with PC1. As shown in Fig. 5B, each variable contributed nearly equally to PC1 (~ 33.3 to 33.7%), suggesting that sample separation along this axis was driven by a balanced contribution of biomass and water-soluble protein production. The wild–type strains HUF16M2K10004 and HUF16M3G11088 were positioned in the positive region of PC1 at 48 h and 72 h, respectively, reflecting their enhanced fermentation performance comparable to that of the commercial reference strain MERIT™, grouped within the same region. In contrast, HUF16M3H11101 was generally located near the origin or in the negative regions of both PC1 and PC2, indicating comparatively low biomass and water-soluble protein production throughout the fermentation period.

Although the present study quantified water–soluble protein (WSP) rather than total intracellular protein, WSP values were expressed on a dry–biomass basis as percentage ratios for comparative interpretation. The wild–type strains HUF16M2K10004 and HUF16M3G11088 yielded water-soluble protein fractions corresponding to 33.35% and 29.73% at 72 h, which were comparable to those obtained under identical conditions for commercial reference strains 9763 (33.87%) and MERIT™ (34.02%). In contrast, HUF16M3H11101 produced a markedly lower water-soluble protein fraction (12.62%), consistent with the autolysis previously discussed. The observed values were within the range reported in previous preliminary SCP screening studies: Bertasini et al. (2022) reported a maximum of 28% (w/w) protein on candy production effluent, whereas Tropea et al. (2022) obtained 33.48–38.43% true protein at 72–120 h on a multi–substrate food waste medium. Dunuweera et al. (2021) reported a broader range of 9.64–48.32% across different fruit waste media, with the highest value observed on pineapple–based substrate. Notably, these studies quantified crude or true protein, typically based on Kjeldahl total nitrogen determination, which is methodologically distinct from the water-soluble protein fraction determined using the Bradford assay in the present study. Therefore, these values are presented for indicative purposes only and should be interpreted within the limited scope of this preliminary screening, rather than as a direct quantitative comparison of total protein content.

In the present study, white grape juice provided a hostile environment that effectively enabled strain screening, underscoring its suitability as a food–grade substrate. The multivariate analyses demonstrated that the wild–type strains HUF16M2K10004 and HUF16M3G11088 consistently achieved WCW, DCW, and WSP contents comparable to those of commercial reference strains, supporting their potential as preliminary candidates for future SCP–oriented characterization studies and contributing to the development of alternative protein sources within circular bioeconomy frameworks. It should be noted that the protein assay method employed in this study recovers the water–soluble protein fraction rather than total intracellular protein and therefore reflects the extractable water-soluble protein fraction rather than total protein content or nutritional quality. As such, the present WSP values are most appropriately interpreted as a comparative screening indicator within the scope of this preliminary study. This approach is consistent with previous SCP studies (Ayodele et al., 2024; Tian et al., 2023); future studies employing complete cell disruption methods (e.g., bead beating or sonication) would allow a more comprehensive assessment of total cellular protein content, alongside amino acid profiling and nutritional characterization of SCP.

In the context of scale–up and industrial SCP production, substrate cost is a critical determinant, accounting for 45–75% of total production costs (Sharif et al., 2021); accordingly, the evaluation of agro–industrial by–products as fermentation substrates—including lower–cost nitrogen sources such as urea, ammonium sulfate, or agro–industrial nitrogen–rich by–products as alternatives to yeast extract—represents an important direction for future process optimization.

## Supplementary Information

Below is the link to the electronic supplementary material.

Supplementary file1 (DOCX 807 kb)
