# Supplementary material for: Screening of wild-type Saccharomyces cerevisiae strains for single-cell protein production in white grape juice
Source: Food Sci Biotechnol. 2026 Jun 30;35(9):2603–12. doi: 10.1007/s10068-026-02215-8 (PMC13421595; doi:10.1007/s10068-026-02215-8)
Supplement: Supplementary file 1 — Supplementary file1 (DOCX 807 kb) [file 10068_2026_2215_MOESM1_ESM.docx]

**Supporting Information**

**Title:** Screening of Wild-Type *Saccharomyces cerevisiae* Strains for Single-Cell Protein Production in White Grape Juice

**Short Title:** Grape Juice Fermentation for SCP Production

İpek Ceren Yeşildağ^1^ and Remziye Yılmaz^[[1]](#footnote-1)*^

^1^Hacettepe University, Department of Food Engineering, FoodOmics Laboratory, 06800, Ankara, Türkiye

**Table S1.** Characterization of wild-type and commercial *Saccharomyces cerevisiae* strains with respect to morphology and growth at pH 3.3 and pH 6.5

**Table S2.** Growth measurements of *S. cerevisiae* 9763, MERIT^TM^, HUF16M2K10004, HUF16M3G11088 and HUF16M3H11101 strains determined by optical density measurement (OD_600_). The values (means ± SD, n = 4) with different letters are significantly different according to the Tukey test, p ˂ 0.05

**Table S3.** Biomass yields in terms of wet cell weight (WCW), dry cell weight (DCW) and growth kinetics (specific growth rate and doubling time) of 9763, MERIT^TM^, HUF16M2K10004, HUF16M3G11088 and HUF16M3H11101 strains in white grape juice medium. Values are presented as means ± SD (n = 3)

**Table S4.** Water-soluble protein content of 9763, MERIT^TM^, HUF16M2K10004, HUF16M3G11088 and HUF16M3H11101 strains in white grape juice medium. Values are presented as mean ± SD (n = 4). Different letters within the same row indicate statistically significant differences among strains at the same time point according to Tukey's post-hoc test (p < 0.05)

**Table S1.** Characterization of wild-type and commercial *Saccharomyces cerevisiae* strains with respect to morphology and growth at pH 3.3 and pH 6.5

| **Source** | **Isolation Code** | **Macroscopic Morphology** | **Microscopic Morphology** | **Colony Characteristics** | **pH 3.3** | **pH 6.5** |
| --- | --- | --- | --- | --- | --- | --- |
| *S. cerevisiae* ATCC 9763 | 9763 | 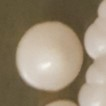 | 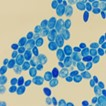 | Ovoid, Smooth, Light cream- colored | ++ | ++ |
| *S. cerevisiae MERIT^TM^  (Chr. Hansen)* | *MERIT^TM^* | 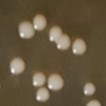 | 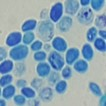 | Ellipsoidal, Shiny and light-cream colored | ++ | ++ |
| *S. cerevisiae* ATCC 6328  HUF | 6328 | 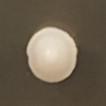 | 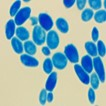 | Ovoid, Smooth, Light cream- colored | ND | ++ |
|  | 16M2K10004 | 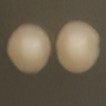 | 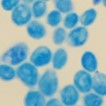 | Ellipsoidal, Smooth, Light cream-colored | ++ | ++ |
|  | 16M3B11021 | 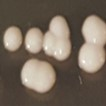 | 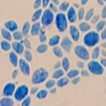 | Ellipsoidal, Shiny and light-cream colored | + | ++ |
|  | 16M3C11032 | 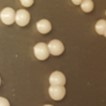 | 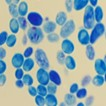 | Ovoid, Shiny and light-cream colored | + | ++ |
|  | 16M3C11033 | 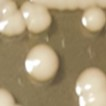 | 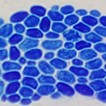 | Ellipsoidal, Shiny and light-cream colored | + | ++ |
|  | 16M3G11088 | 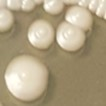 | 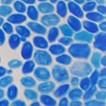 | Ellipsoidal, Smooth, Light cream-colored | ++ | ++ |
|  | 16M3H11101 | 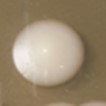 | 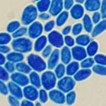 | Ellipsoidal, Smooth, Light cream-colored | ++ | ++ |
|  | 17M3C31063 | 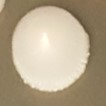 | 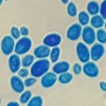 | Ovoid, Smooth, Light cream- colored | + | ++ |
|  | 17M3D31088 | 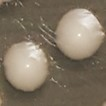 | 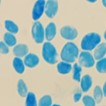 | Ellipsoidal, Smooth, Light cream-colored | + | ++ |
|  | 17M3E21113 | 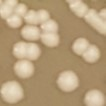 | 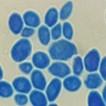 | Ellipsoidal, Shiny and light-cream colored | + | ++ |
|  | 17M3E21114 | 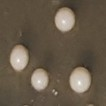 | 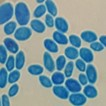 | Ellipsoidal, Smooth, Light cream-colored | + | ++ |
|  | 17M3F21122 | 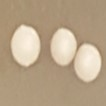 | 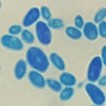 | Ellipsoidal, Smooth, Light cream-colored | + | ++ |
|  | 17M3H21208 | 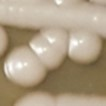 | 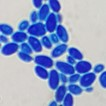 | Ellipsoidal, Shiny and light-cream colored | + | ++ |
|  | 17M3H21209 | 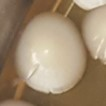 | 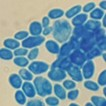 | Ellipsoidal, Smooth, Light cream-colored | + | ++ |
|  | 18M2Y10013 | 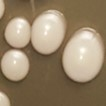 | 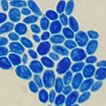 | Ellipsoidal, Smooth, Light cream-colored | + | ++ |
|  | 18M2Z10001 | 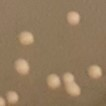 | 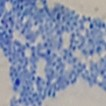 | Ellipsoidal, Smooth, Light cream-colored | + | ++ |

++, Robust visible growth; +, Weak visible growth; ND: Not determined.

**Table S2.** Growth measurements of *S. cerevisiae* 9763, MERIT^TM^, HUF16M2K10004, HUF16M3G11088 and HUF16M3H11101 strains determined by optical density measurement (OD_600_). The values (means ± SD, n = 4) with different letters are significantly different according to the Tukey test, p ˂ 0.05

|  | **OD_600_** | | | | |
| --- | --- | --- | --- | --- | --- |
| **Time (h)** | **9763** | **MERIT^TM^** | **HUF16M2K10004** | **HUF16M3G11088** | **HUF16M3H11101** |
| 0 | 0 ± 0^g^ | 0.05 ± 0.00^d^ | 0.01 ± 0.06^e^ | 0.04 ± 0.01^e^ | 0.02 ± 0.02^f^ |
| 10 | 0 ± 0^g^ | 0.14 ± 0.12^cd^ | 0.33 ± 0.08^d^ | 0.04 ± 0.01^e^ | 0.19 ± 0.20^f^ |
| 12 | 0.08 ± 0.01^f^ | 0.08 ± 0.01^cd^ | 0.54 ± 0.06^cd^ | 0.09 ± 0.02^e^ | 0.72 ± 0.06^e^ |
| 14 | 0.18 ± 0.01^e^ | 0.15 ± 0.01^cd^ | 0.76 ± 0.03^c^ | 0.15 ± 0.01^de^ | 0.97 ± 0.03b^cd^ |
| 16 | 0.34 ± 0.02^d^ | 0.29 ± 0.01^bc^ | 1.03 ± 0.04^b^ | 0.26 ± 0.05^d^ | 1.17 ± 0.02^ab^ |
| 18 | 0.57 ± 0.04^c^ | 0.52 ± 0.01^b^ | 1.25 ± 0.05^b^ | 0.46 ± 0.06^c^ | 1.29 ± 0.06^a^ |
| 20 | 0.73 ± 0.02^b^ | 0.99 ± 0.27^a^ | 1.23 ± 0.12^b^ | 0.57 ± 0.08^c^ | 1.11 ± 0.02^abc^ |
| 22 | 0.91 ± 0.06^a^ | 0.83 ± 0.04^a^ | 1.52 ± 0.16^a^ | 0.98 ± 0.07^b^ | 0.94 ± 0.14^cd^ |
| 24 | 0.93 ± 0.01^a^ | 0.82 ± 0.01^a^ | 1.67 ± 0.15^a^ | 1.21 ± 0.08^a^ | 0.87 ± 0.04^de^ |

**Table S3.** Biomass yields in terms of wet cell weight (WCW), dry cell weight (DCW) and growth kinetics (specific growth rate and doubling time) of 9763, MERIT^TM^, HUF16M2K10004, HUF16M3G11088 and HUF16M3H11101 strains in white grape juice medium. Values are presented as mean ± SD (n = 3)

|  | **Wet Cell Weight (g/L)** | | | | |
| --- | --- | --- | --- | --- | --- |
| **Time (h)** | **9763** | **MERIT^TM^** | **HUF16M2K10004** | **HUF16M3G11088** | **HUF16M3H11101** |
| 24 | 9.25 ± 1.30 | 6.20 ± 0.20 | 5.35 ± 0.60 | 7.29 ± 2.70 | 8.55 ± 1.40 |
| 48 | 17.97 ± 1.06 | 10.51 ± 0.65 | 19.75 ± 1.65 | 22.34 ± 0.46 | 10.69 ± 0.77 |
| 72 | 15.21 ± 0.57 | 19.35 ± 0.26 | 21.56 ± 0.13 | 27.90 ± 0.49 | 18.58 ± 0.89 |
| **Time (h)** | **Dry Cell Weight (g/L)** | | | | |
| 24 | 1.40 ± 0.12 | 0.78 ± 0.09 | 0.60 ± 0.02 | 1.01 ± 0.49 | 1.30 ± 0.30 |
| 48 | 4.68 ± 0.34 | 3.12 ± 0.43 | 5.65 ± 0.95 | 5.30 ± 0.84 | 2.40 ± 0.26 |
| 72 | 4.77 ± 0.70 | 5.82 ± 0.71 | 6.01 ± 0.05 | 6.46 ± 0.44 | 5.02 ± 0.12 |
|  | **Growth Kinetics** | | | | |
| **Growth Rate** | 0.29 ± 0.02 | 0.31 ± 0.06 | 0.15 ± 0.02 | 0.23 ± 0.02 | 0.11 ± 0.03 |
| **Doubling Time** | 2.39 ± 0.15 | 2.36 ± 0.51 | 4.90 ± 0.53 | 3.08 ± 0.23 | 6.72 ± 1.58 |

**Table S4.** Water-soluble protein content of 9763, MERIT^TM^, HUF16M2K10004, HUF16M3G11088 and HUF16M3H11101 strains in white grape juice medium. Values are presented as mean ± SD (n = 4). Different letters within the same row indicate statistically significant differences among strains at the same time point according to Tukey's post-hoc test (p < 0.05)

|  | **Water-Soluble Protein (g/L)** | | | | |
| --- | --- | --- | --- | --- | --- |
| **Time (h)** | **9763** | **MERIT^TM^** | **HUF16M2K10004** | **HUF16M3G11088** | **HUF16M3H11101** |
| 24 | 0.99 ± 0.05^a^ | 0.38 ± 0.04^c^ | 0.38 ± 0.04^c^ | 0.58 ± 0.01^b^ | 0.62 ± 0.05^b^ |
| 48 | 1.61 ± 0.11a^b^ | 1.79 ± 0.22^a^ | 1.29 ± 0.07^bc^ | 1.69 ± 0.19^ab^ | 1.10 ± 0.33^c^ |
| 72 | 1.62 ± 0.01^a^ | 1.98 ± 0.10^b^ | 2.00 ± 0.15^ab^ | 1.92 ± 0.07^b^ | 0.63 ± 0.11^c^ |

1. * Corresponding author: Tel: +90 312 297 71 06, Fax: +90 312 299 21 23

   E-mail address: [remziye@hacettepe.edu.tr](mailto:remziye@hacettepe.edu.tr) , [remziye06@gmail.com](mailto:remziye06@gmail.com) [↑](#footnote-ref-1)
